# Changes in melanocytic nevi after treatment with intense pulsed light observed in total body mapping*

**DOI:** 10.1590/abd1806-4841.20165389

**Published:** 2016

**Authors:** Maria Fernanda Vianna Hunziker, Eduardo Botelho Silva Mauad, Luís Fernando Amarante Fernandes, Ana Maria Costa Pinheiro

**Affiliations:** 1 Universidade de Brasília (UnB) – Brasília (DF), Brazil

Intense Pulsed Light (IPL) is a broadband visible light emitted from a non-coherent, filtered flashlamp. IPL sources emit light in the 500-1200 nm range and allow treatment of vascular and melanocytic lesions, and also hair removal. Different cutoff filters can be used to narrow the spectrum to target specific structures.^1^

We report significant changes observed in two nevi after hair removal treatment with IPL. One of them underwent complete regression.

A 35-year-old woman with multiple acquired melanocytic nevi was referred to our clinic for complete body evaluation using digital dermoscopy. The exam revealed some significant changes in two nevi located on her legs. Dermoscopy showed several blotches of brownish pigment without any pattern of a melanocytic lesion.

Anamnesis revealed that the patient had undergone an IPL session to remove hair from her legs a few weeks earlier. The patient had not realized that the nevi had changed.

**Figure 1:**
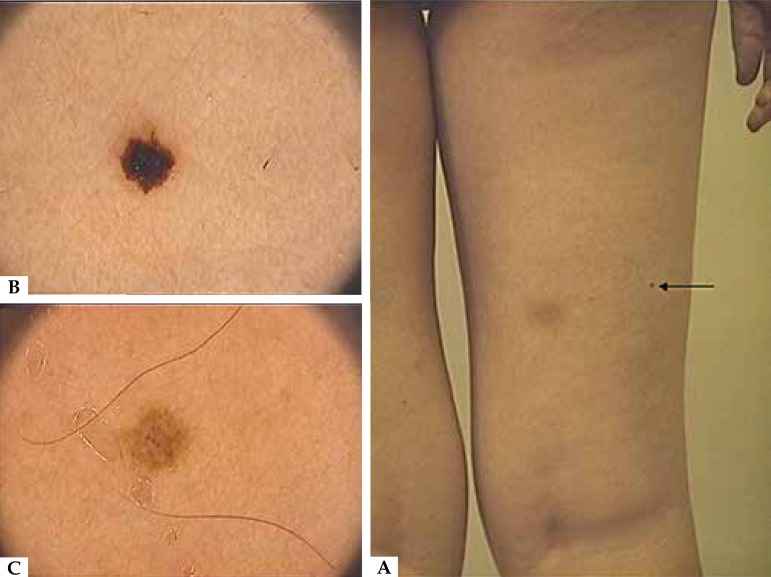
**a)** Posterior side of the right leg with a melanocytic lesion (arrow); **b)** First dermoscopy image of the melanocytic lesion, showing brownish blotches; **c)** Image of the same lesion after a 12-week C A period

**Figure 2:**
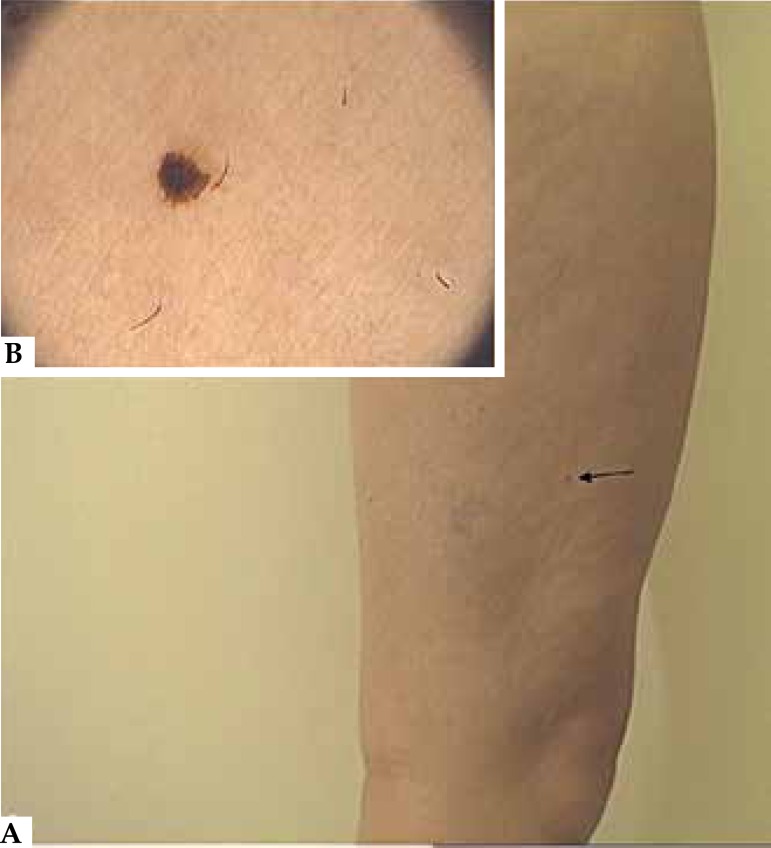
**a)** Lateral aspect of the right leg. **b)** Dermoscopic image of the nevus indicated by the arrow in "a". There is a homogeneous brownish blotch

**Figure 3:**
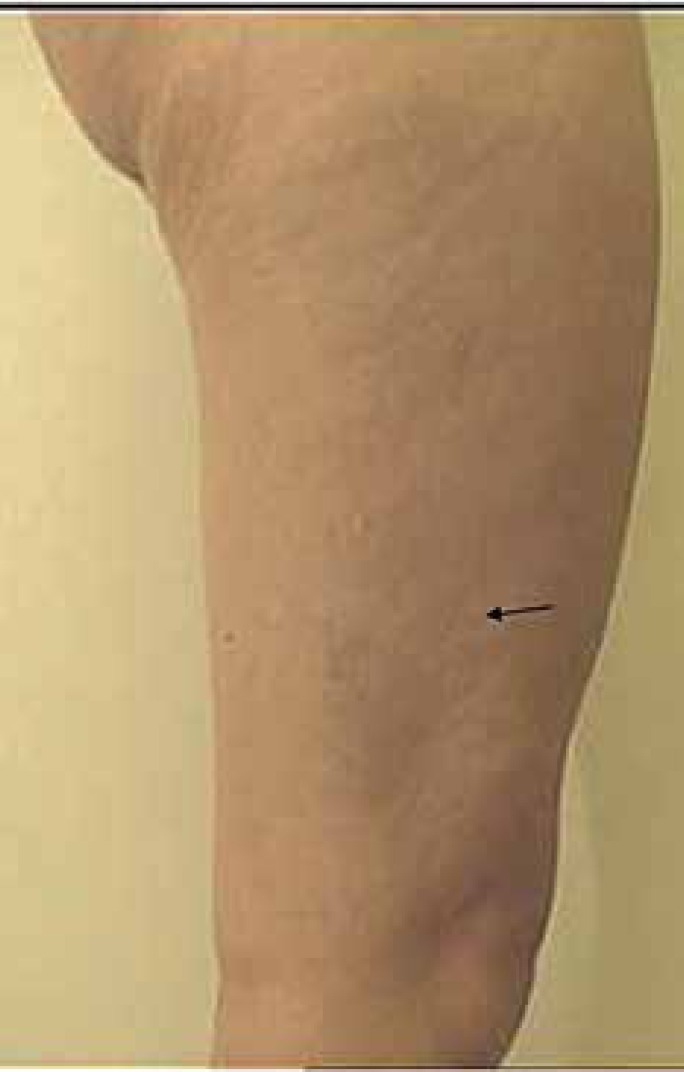
Lateral aspect of the right leg, after a 12-week follow-up period. The nevus seen in Figure 2 is no longer present (arrow)

We decided to observe and follow-up the patient. A new dermoscopy was conducted three months later. The nevus on the posterior side of her right leg showed smaller brownish spots on a reticulate pattern (Figure 1). Meanwhile, the nevus located on the lateral side of her right leg had disappeared (Figures 2 and 3).

The brownish blotches seen in dermoscopy images of the two nevi of our patient correspond to microcrusts observed after IPL therapy. ^2,3^ Electron microscopy studies of the microcrusts formed 2 to 5 days after IPL sessions revealed multiple melanosomes and cellular debris. IPL therapy is reported to be effective for treating some pigmented lesions. The theory of selective photothermolysis explains how the controlled absorption of thermal energy by targeted chromophores lead to their destruction without significant thermal damage to the surrounding tissue. ^2^

A complete regression of melanocytic nevus after IPL therapy was also described by Martín *et al.*, where the initial lesion was reticular-patterned on dermoscopy and after IPL session it showed multiple blotches of brownish pigment, similar to that seen in our patient. The lesion was removed and histology showed a microcrust in the epidermis above the papillary dermis with delicate fibrosis and a mild superficial inflammatory component with melanophages. ³

IPL and melanocyte-target lasers can eliminate superficial melanocytes. ^2,3^ However, complete regression of melanocytic nevus after IPL or laser treatment, as occurred in this case, is uncommon. In most cases, repigmentation is frequently seen. ^2,3,4^ These therapies can also cause some other modifications, such as asymmetric pattern, blue-gray dots, and milky red veil. Incomplete treatment of a melanocytic lesion may result in a regression that resembles clinically and histologically a superficial spreading melanoma. ^5^ The term pseudomelanoma can describe these lesions. ^5^ Despite the changes induced by light systems, further studies are necessary to discard the risk of malignant transformation and whether or not to advice against these procedures in patients with high risk of melanoma.

In this case, after a 12-week follow-up period, the dermoscopic characteristics of one lesion (as shown in Figure 1) supported the diagnosis of a benign melanocytic lesion. The other one, however, had completely regressed. We recommended to the patient regular clinical and dermoscopic evaluations to observe possible alterations or repigmentation of this lesion. The presence of microcrusts should help in the diagnosis of a pulsed light or laser-induced regression of melanocytic lesion. ^2^

Melanocytic lesions should not be considered a routine indication for laser or IPL therapy. Laser treatment of melanomas with benign clinical features may delay the diagnosis or make it more difficult. Moreover, repigmentation is frequently seen after treatment of melanocytic lesions with lasers or IPL, sometimes with altered morphology, challenging the diagnosis. The delay or error in diagnosis in this scenario often occurs because the pathologist does not know that the pigmented lesion has undergone some local destructive treatment and, therefore, he/she diagnosis malignant melanoma instead of pseudomelanoma. ^2,4^

Dermatology has an important role in the academic medicine and in the diagnosis and management of skin cancer. Dermatologists should focus on skin cancer in their private practice to avoid misdiagnosis of pigmented lesions. Although there are several articles describing the efficacy of lasers in the treatment of pigmented lesions, the first-choice treatment of equivocal skin lesions remains the excision with histopathologic examination.
